# The stability of long-segment and short-segment fixation for treating severe burst fractures at the thoracolumbar junction in osteoporotic bone: A finite element analysis

**DOI:** 10.1371/journal.pone.0211676

**Published:** 2019-02-04

**Authors:** Yueh Wu, Chia-Hsien Chen, Fon-Yih Tsuang, Yi-Cheng Lin, Chang-Jung Chiang, Yi-Jie Kuo

**Affiliations:** 1 Department of Orthopedic Surgery, Taipei Municipal Wanfang Hospital, Taipei Medical University, Taipei, Taiwan; 2 Department of Orthopedics, Shuang Ho Hospital, Taipei Medical University, New Taipei City, Taiwan; 3 Department of Orthopedic Surgery, School of Medicine, College of Medicine, Taipei Medical University, Taipei, Taiwan; 4 Graduate Institute of Biomedical Materials and Tissue Engineering, College of Biomedical Engineering, Taipei Medical University, Taipei, Taiwan; 5 Division of Neurosurgery, Department of Surgery, National Taiwan University Hospital, Taipei, Taiwan; 6 Department of Traumatology, National Taiwan University Hospital, Taipei, Taiwan; 7 Institute of Biomedical Engineering, National Taiwan University, Taipei, Taiwan; Virginia Tech, UNITED STATES

## Abstract

The majority of compressive vertebral fractures in osteoporotic bone occur at the level of the thoracolumbar junction. Immediate decompression is often required in order to reduce the extent of neurological damage. This study evaluated four fixation methods for decompression in patients with thoracolumbar burst fractures, and presented the most suitable method for osteoporotic patients. A finite element model of a T7–L5 spinal segment was created and subjected to an L1 corpectomy to simulate a serious burst fracture. Five models were tested: a) intact spine; 2) two segment fixation (TSF), 3) up-three segment fixation (UTSF), below-three segment fixation (BTSF), and four segment fixation (FSF). The ROM, stiffness and compression ratio of the fractured vertebra were recorded under various loading conditions. The results of this study showed that the ROM of the FSF model was the lowest, and the ROMs of UTSF and BTSF models were similar but still greater than the TSF model. Decreasing the BMD to simulate osteoporotic bone resulted in a ROM for the four instrumented models that was higher than the normal bone model. Of all models, the FSF model had the highest stiffness at T12-L2 in extension and lateral bending. Similarly, the compression ratio of the FSF model at L1 was also higher than the other instrumented models. In conclusion, FSF fixation is suggested for patients with osteoporotic thoracolumbar burst fractures. For patients with normal bone quality, both UTSF and BTSF fixation provide an acceptable stiffness in extension and lateral bending, as well as a favorable compression ratio at L1.

## Introduction

Burst fractures of the thoracolumbar segment in the spine typically occur where the lowest thoracic vertebrae connects to the first lumbar vertebrae. A burst fracture occurs when an axial compressive force on the anterior and middle column collapses the bone and causes failure of the anterior and middle supporting columns [1]. The thoracolumbar segment is the most common site for unstable burst fractures, representing approximately 15% of vertebral injuries [2].

There is no general consensus on the most suitable method for treating thoracolumbar burst fractures. For severely unstable fractures or in the event of impaired neurologic function, surgical repair is necessary. Stabilization of the fracture using an anterior approach can decompress any impinged nerves, while a strut made of an iliac graft can support the collapsed vertebral body. The entire construct can then be fixed with a locked thoracolumbar plate. Hitchon et al. [3] reported on the outcomes of patients with T11-L2 thoracolumbar burst fractures that underwent decompression using an anterolateral or posterior reconstruction approach. The results indicated that the anterior approach was superior to the posterior approach in correcting and maintaining an acceptable kyphotic curve. However, the posterior approach was found to be easier to perform, resulted in less trauma and blood loss, was more cost-effective, demonstrated better recovery of neurological function and had superior canal decompression [4]. Hence, stabilization with posterior pedicle screws is the most common approach today for the repair of thoracolumbar burst fractures [5,6].

Although short-segment pedicle screw stabilization (one-level above and below the fracture level) is typically considered to provide enough stability for thoracolumbar burst fractures, the reported failure rate is quite high [7,8]. Hence, vertebroplasty or bone grafts have been used to supplement the fracture site in order to maintain the anterior column and reduce the incidence of short-segment failure [9,10]. However, some studies have indicated that anterior column augmentation can lead to cement leakage and the unpredictable displacement of bone fragments, and these procedures often cannot achieve full reduction of kyphosis [11–13].

In contrast to short-segment pedicle screw stabilization, long-segment posterior fixation (two-levels above and below the fracture level) offers greater stability and a more effective reduction in kyphotic deformities [14,15]. A meta-analysis study [11] indicated that long-segment fixation could offer superior results in terms of radiographic indexes and implant failure, but the clinical outcome suggested that there was no significant difference between the two groups. Long-segment fixation also requires a greater number of vertebrae, which significantly extends the length of the immobile segment in the spine.

As a compromise, three-level stabilization (two-levels above and one-level below the fracture level) for fractures at the thoracolumbar junction in combination with short segment fixation in the lumbar area can provide sufficient stability to the spine, reduce the length of the immobile segment, and reduce the incidence of kyphotic collapse [16,17]. To date, no biomechanical study has been performed to quantify the stability of posterior two-, three- and four-segment fixation. The purpose of this current study is to analyze the effect of treating thoracolumbar burst fractures with two-, three- and four-segment fixation using finite element models.

## Methods

**Fig 1:**
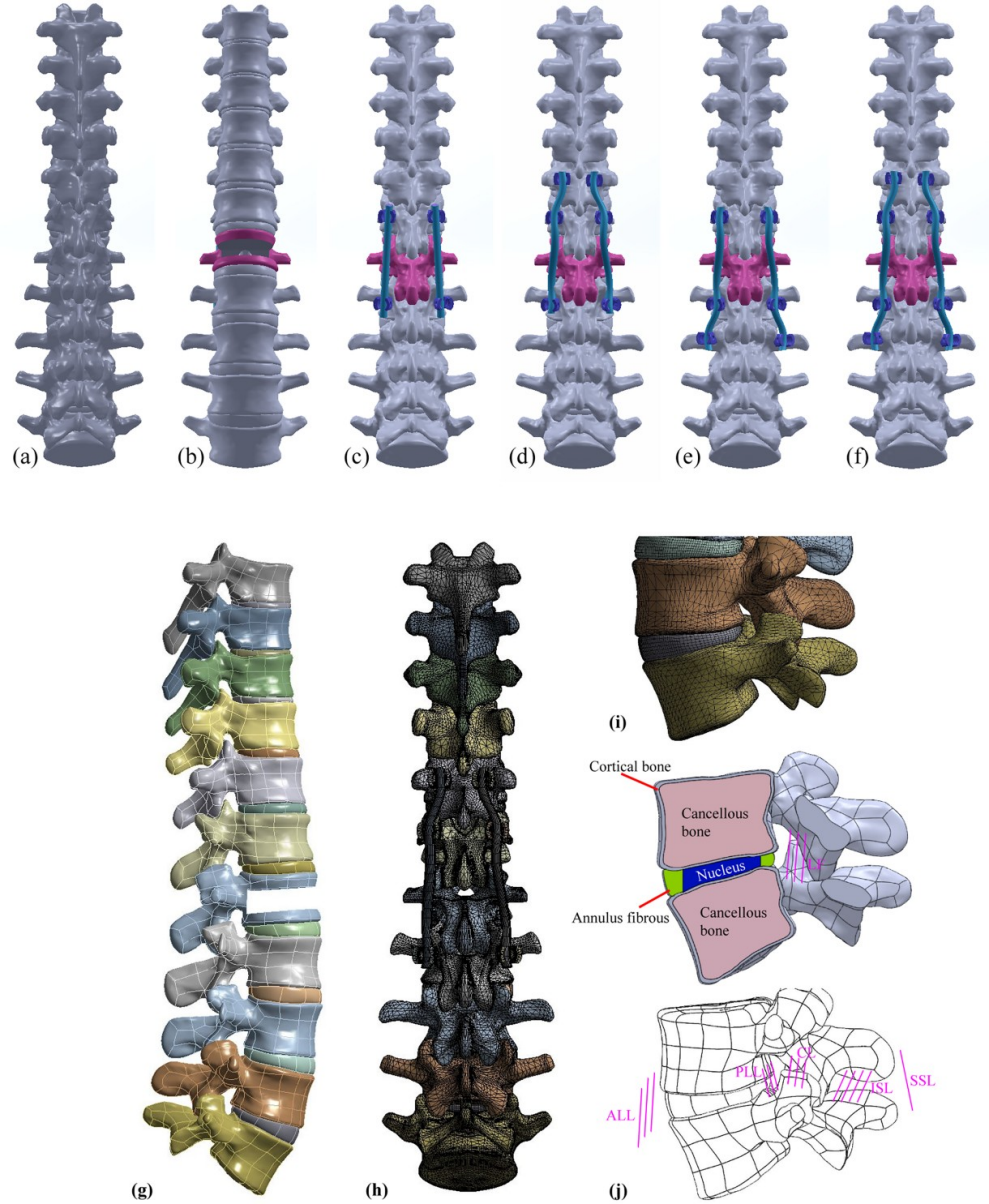
**Posterior view of T7-L5 spine models:** a) intact; b) L1 corpectomy performed to simulate serious burst fracture; c) two-segment fixation at T12-L2 (TSF); d) up-three segment fixation at T11-L2 (UTSF); e) below-three segment fixation at T12-L3 (BTSF); f) four-segment fixation at T11-L3 (FSF); g) burst fracture segment at L1 to simulate a severe wedge deformity (Grade 3) [23]; h) and i) finite element mesh of T7-L5; and j) L4–L5 finite element model containing anterior longitudinal ligament (ALL), posterior longitudinal ligament (PLL), ligamentum flavum (LF), interspinous ligament (ISL), supraspinous ligament (SSL), and capsular ligaments (CL).

The finite element software ANSYS 16.0 (ANSYS Inc., Canonsburg, PA, USA) was used to create an FE model of an 11-level thoracolumbar spine. As shown in Fig 1A, a T7–L5 spine segment was developed using geometry from a morphologically accurate spinal model that included vertebrae and intervertebral discs (Zygote Media Group, Inc.). The annulus material was based on an incompressible, hyperelastic, 2-parameter (C1, C2) Mooney-Rivlin formulation, and the nucleus pulposus was modeled as an incompressible fluid. The anterior longitudinal ligament, posterior longitudinal ligament, ligamentum flavum, interspinous ligament, supraspinous ligament, and capsular ligaments were assigned properties based on published experimental values and approximated as nonlinear, tension-only springs (ANSYS 16.0) with insertion points approximated to typical anatomy [18,19]. These ligaments were represented as 2-node tension-only link elements, as shown in Fig 1J. The material properties of the T7–L5 model were sourced from literature [18–22] and are shown in Table 1.

**Table 1:** Material properties for T7–L5 spine segment model.

Property	Modulus (MPa)	ν	References
Cortical bone	12,000	0.2	Goel et al., 1995 [21]
Cancellous bone (Normal/Osteoporosis)	300/100	0.2	Morgan et al., 2003 [22] Liu et al., 2014 [28]
Annulus fibrous	Mooney-Rivlin c1 = 0.18, c2 = 0.045	NA	Schmidt et al., 2007 [18]
Nucleus pulposus	Mooney-Rivlin c1 = 0.12, c2 = 0.03	NA	Schmidt et al., 2007 [18]
Ligaments	Hyperelastic	NA	Schmidt et al., 2007 [18]
Titanium spinal rods and pedicle screws	110,000	0.3	Li et al., 2014 [35]

NA = not applicable

Five finite element models were developed in this study: a) intact thoracolumbar spine (INT) without any implants (Fig 1A), b) thoracolumbar spine with an L1 corpectomy to simulate a Grade 3 wedge deformity [23] from a burst fracture (Fig 1B and Fig 1G) and implanted with a posterior spinal fixator c) two-segment fixation at T12-L2 (TSF, Fig 1C), d) up-three segment fixation at T11-L2 (UTSF, Fig 1D), e) below-three segment fixation at T12-L3 (BTSF, Fig 1E), and four-segment fixation at T11-L3 (FSF, Fig 1F). Titanium spinal rods and pedicle screws were incorporated into a CB PROT II Posterior Spinal System (Chin Bone Corp., Taiwan; US FDA 510(k): K142655), which consists of titanium alloy screws (diameter 5.5 mm) connected by vertical rods (diameter 5.5 mm). The material properties of the implants are shown in Table 1. All implant components (pedicle screws and titanium spinal rods), cortical bone, cancellous bone and disc were modeled using 8-node solid elements. For the disc, twelve double cross-linked fibrous layers were embedded in the ground substance, and fiber stiffness increased proportionally from the outermost layer to the innermost layer. The nucleus pulposus was modeled as an incompressible fluid using 8-node fluid elements. All spine models were meshed with a combination of tetrahedral elements for the vertebrae and hexahedral elements for the intervertebral discs (Fig 1H and Fig 1I). The entire model consisted of approximately 290,400 elements and 616,500 nodes.

The interfaces between facet articular surfaces were treated as standard contact pairs at all levels. The spinal fusion segment was defined by multiple adjacent vertebrae bridged with pedicle screws and rods. In addition, the interfaces between the spinal rods, pedicle screws and bone were bonded. An unconstrained pure moment of 5.0 Nm was applied to the superior endplate of T7. The distal vertebra was restricted from all motion by rigidly anchoring the inferior endplate of L5, effectively acting as a fusion to the pelvis.

The model was successfully validated by comparing segmental stiffness with experimental in vitro data and finite element analysis results from literature [18,19, 24–27]. An unconstrained pure flexion moment of 7.5 Nm was applied in four directions (flexion, extension, lateral bending, axial rotation) to the superior endplate of T7, and the distal vertebra was restricted from all motion by rigidly anchoring the inferior endplate of L5. The range of motion of the segment was recorded and found to be within the ranges reported from in vitro studies (Table 2).

**Table 2:** Range of motion (ROM) of intact spinal models from literature and this study.

	Applied pure moment (Nm)	ROM (segment)	Flexion (degree) ±SD	Extension (degree) ±SD	Lateral bending (degree) ±SD	Axial rotation (degree) ±SD
Niosi et al., 2006 **[25]**	7.5	L3-L4	3.7(±1.5)	3.3(±1.5)	3.8(±1.4)	2.1(±0.9)
Panjabi et al., 1994 **[43]**	7.5	L3-L4	6.5	2.0	5.0/4.5	1.8/2.0
Schilling et al., 2011 **[26]**	7.5	L3-L4	4.67(±1.79)	2.18(±0.54)	7.66(±2.91)	4.67(±2.52)
*Our study	7.5	L3-L4	3.9	2.4	4.7	2.2
*Schilling et al*., *2011* **[26]**	*7*.*5*	*L4-L5*	*5*.*62(±2*.*17)*	*3*.*32(±1*.*12)*	*7*.*76(±1*.*85)*	*5*.*16(±1*.*30)*
*Our study	*7*.*5*	*L4-L5*	*5*.*1*	*3*.*1*	*4*.*8*	*3*.*9*

This study analyzed the range of motion (ROM) and stiffness across the T12 and L2 vertebrae under flexion, extension, torsion, and left lateral bending. In addition, the anterior body height of L1 was modeled with both normal bone and osteoporotic bone and was placed under flexion bending. The material properties of osteoporotic cancellous bone (age greater than 75 years old) were sourced from literature [28]. The range of motion of the segment was also recorded and found to be within the ranges reported from an in vitro study [29].

## Results

### ROM of each model between T12-L2

**Fig 2:**
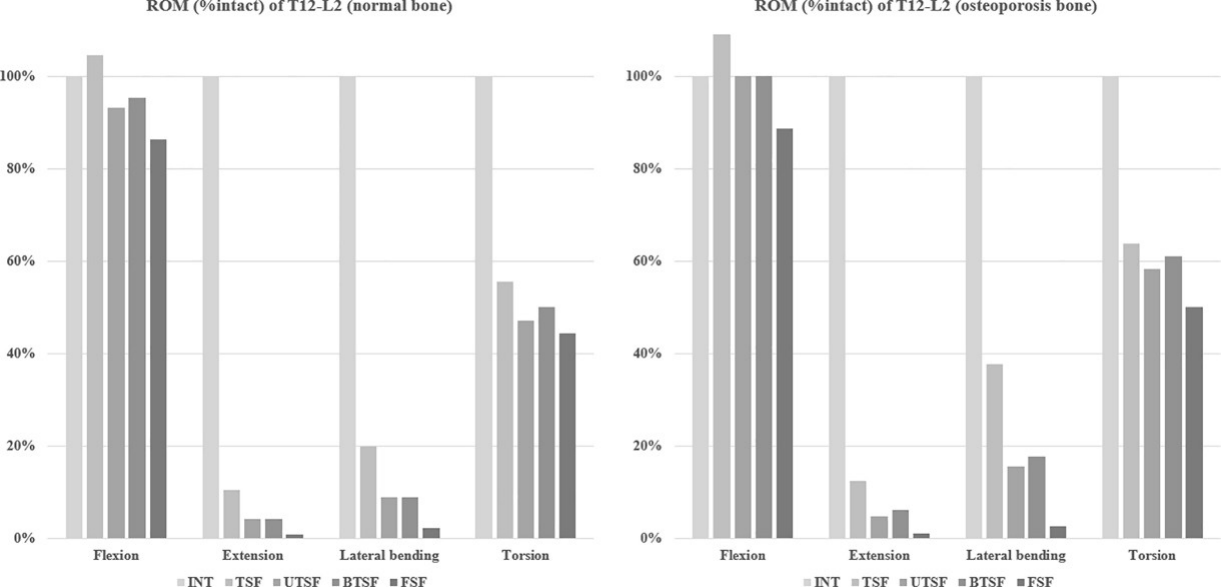
ROM of T12-L2 in each model normalized by the ROM of INT.

Fig 2 shows that the ROM decreased around the fusion site at T12-L2 in all implanted models except for the TSF model in flexion. In comparison to the osteoporotic models, the ROM was greater than the models with normal bone. The ROM of the TSF, UTSF, BTSF, and FSF models (either with normal or osteoporotic bone) was significantly lower than the intact (INT) model in extension, lateral bending and torsion.

### Stiffness of each model at T12-L2 segment

**Fig 3:**
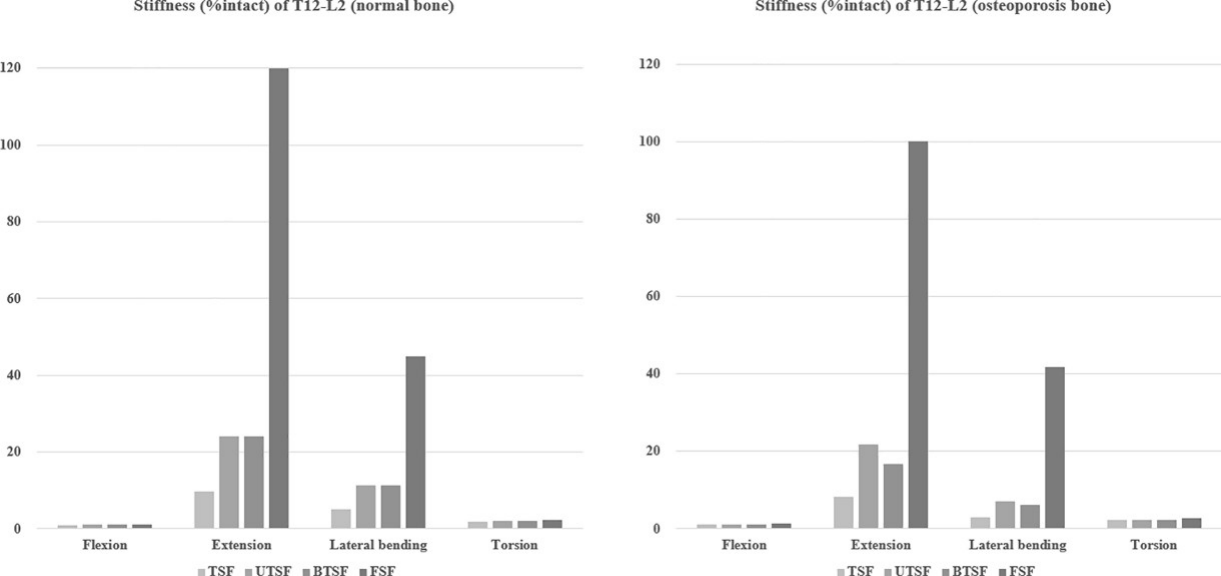
Stiffness of T12-L2 in each model normalized by the stiffness of INT.

Fig 3 shows that the stiffness increased at T12-L2 in all implanted models except for in the TSF model in flexion. In comparison to the models with osteoporosis bone, the stiffness was lower than the models with normal bone. The stiffness of the TSF, UTSF, BTSF, and FSF models (either with normal or osteoporotic bone) was significantly higher than the intact (INT) model in extension and lateral bending. In particular, the stiffness of the FSF model was noticeably greater that the INT and other fusion models.

### Anterior body height of L1

**Fig 4:**
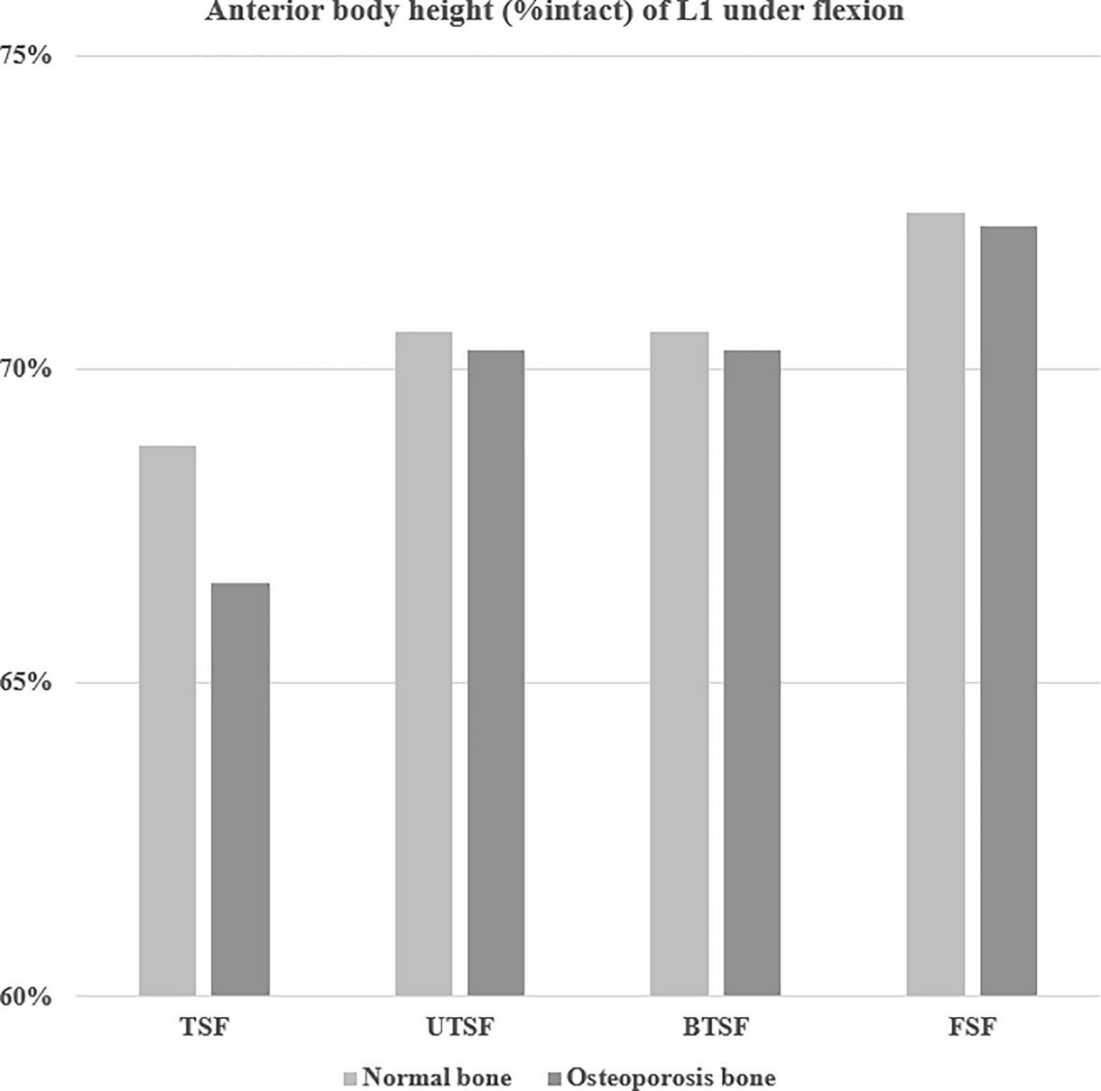
Anterior body height of L1 under flexion in each model normalized by the anterior body height of INT.

Fig 4 shows the compression ratio of anterior body height at L1 at the fusion site in all implanted models under flexion. The compression ratio of the FSF model was highest among all models, whether with normal or osteoporotic bone. The compression ratio was similar between the UTSF and BTSF models.

## Discussion

The purpose of pedicle screw stabilization is to maintain spinal stability to facilitate bone healing. But the high reported failure rates of instrumented segments, leading to traumatic instability, has led to an unacceptable incidence of anterior column defects [30]. The thoracolumbar junction is a transition zone between the posterior thoracic curve and the anterior lumbar curve and experiences some of the highest stress levels in the spine. This has resulted in a high incidence of burst fractures in the region when compared to other areas of the thoracic or lumbar spine [31]. With advances in implant materials and manufacturing technologies, the success rate of fixation screws is improving and surgeons are increasingly opting for short-segment fixation. Minimizing the number of vertebral segments required for fixation is also an important goal of internal fixation in order to maintain flexibility. However, failure rates of between 20% and 50% have been reported with the use of short-segment fixation for thoracolumbar burst fractures [32–34]. Hence, the objective of this finite element study was to investigate the importance of the number of fixed segments used for treating thoracolumbar burst fractures.

The angular ROM of each model was in good agreement with published in-vitro studies [18,19, 24–27], as shown in Tables 1 and 2. This study showed that long-segment fixation is stiffer than short-segment fixation, with the lower ROM in extension, bending, and torsion being consistent with previous finite element studies [35,36]. However, when placed in flexion, the short-segment construct had a greater ROM than the intact spine model [35,36]. A possible reason is the different location of the fracture site. In this current study, 60% of the middle region of the L1 segment was resected, and the structure of the posterior part was slightly reserved to establish a finite element model of an unstable thoracolumbar fracture. This structure was weaker than other studies [35,36].

When modeled with both normal bone density and osteoporotic bone density, the TSF model showed a greater ROM than all other fixed models for each of the loading conditions. Notably, for flexion movements, the TSF model permitted a greater ROM than the intact model (105% with normal bone density and 109% with osteoporotic bone). In the other words, vertebrae fixed with UTSF, BTSF, and FSF were more rigid and stable, likely due to the fact that these fixation methods employed a greater number of segments. The FSF group offered the greatest mechanical stiffness, signifying that this method may be used as a fusion technique to prevent segmental collapse. However, when such rigid implants limit spinal movements over an extended period of time, this may lead to adjacent segment disease due to the excessive motion of adjacent levels [37]. Although FSF was shown to offer superior mechanical stability and stiffness, in contrast, it is also thought to require a longer operation time, and result in greater blood loss and soft tissue damage.

There was no significant difference in ROM and stiffness between the UTSF and BTSF models, and both models demonstrated a lower ROM than the TSF and INT models under all loading condition. The addition of one adjacent level led to a stronger 3-point posterior support than the TSF construct. Anekstein et al. [38] indicated that the addition of posterior fixation points could significantly increase the stiffness of pedicle screw fixation for burst fractures and more fixation points could theoretically reduce the stress on the individual instrument components. Canbek et al. [39] recorded data from 25 consecutive patients to compare the radiological and functional results between UTFS and FSF for the treatment of thoracolumbar burst fractures and failed to find any significant differences between the two groups in terms of long-term functional and radiographic results. In this study, when placed under the flexion, extension, lateral bending, and rotation moments, the ROM of the UTSF model was 14%, 3.5%, 8.2% and 3% greater than the FTF model with normal bone density model. In the osteoporotic model, the ROM of UTSF was 11%, 3%, 14% and 8% greater than FSF under flexion, extension, lateral bending, and rotation moments. In addition, anterior body compression following UTSF and FSF was 70.6% and 72.5%, respectively, in the normal bone density model. Similar results were also observed in osteoporotic bone. In summary, FSF fixation did not demonstrate any significant benefits for preventing vertebral collapse over other fixations.

Both UTSF and BTSF resulted in an increase in segmental stiffness. However, physiological loading on the spine occurs in the cranial to caudal direction, so the addition of a single motion segment on the cranial side of the fracture site could alleviate stress in the early stages of healing and help protect the injured vertebrae. Following fixation, the thoracic segments are relatively immobile compared to the lower lumbar spine, so the addition of a single motion thoracic segment may reduce the ROM of the spine and prevent more function of the lower lumbar spine [39,40].

This study also examined the effects of changes in bone mineral density by simulating implantation in osteoporotic bone. The results showed an increase in ROM for all four models in comparison to implantation in normal bone. When placed in flexion, fixation with TSF, UTSF and BTSF could not provide enough stability to the fixation segments, which resulted in an excessive ROM. This was especially pronounced in the TSF model whereby the ROM was greater than the intact model. Similarly, Schulze et al. [41] reported significant migration of pedicle screws following fixation of osteoporotic vertebrae placed under flexion/extension cyclic loading. This is still a challenge in orthopedic surgery today, to achieve proper correction of spinal curvature and prevent screw loosening. Some studies have advocated the placement of pedicle screws in at least two segments above and below the fracture level [15,42]. The results of this current study compliment the use of long-segment fixation in osteoporotic thoracolumbar burst fractures. Future work may involve a clinical, randomized, controlled study to evaluate the reliability of the models presented in this study.

### Limitation of this study

There are some limitations to this study that should be noted.

- This study simulated the treatment of a specific single-level burst fracture. Multi-levels fractures were not analyzed.
- The structure of the vertebral body was assumed as isotropic and homogenous.
- The models did not consider the mechanical effect of muscle contraction, so truly physiological loading was not incorporated into this analysis. The structure of the spine and mechanics of fractures are complex mechanisms that would require a great deal of time and computing power to simulate in detail. The models in this study were simplified to incorporate the primary structures at play in the treatment and stabilization of burst fractures.
- An anterior burst fracture was simulated in this study by removing specific elements from the models. However, most thoracolumbar burst fractures are combined with injury to adjacent segments, which was not considered in this study.
- The cancellous bone quality was only defined by its elastic modulus.
- The data from the finite element models presented in this study represents a clinical tendency, but does not consider individual physiological differences that may be present in clinical practice.

## Conclusion

There is no single "gold standard" method for treating thoracolumbar burst fractures, as a number of aspects such as the bone quality and severity of the fracture should be considered before deciding on a treatment method. This study developed models to simulate severe thoracolumbar burst fractures in both normal bone and osteoporotic bone. The results indicated that FSF fixation was the better choice for osteoporotic bone, probably because it provides the greatest mechanical stiffness for initial fixation and can reduce the likelihood of segmental collapse. However, it may also lead to adjacent segment disease in the long term. Both UTSF and BTSF fixation were acceptable options for normal bone. Particularly in patients with normal bone quality that need a greater ROM, UTSF and BTSF fixation provide an acceptable stiffness in extension and lateral bending, as well as a favorable compression ratio at L1.
